# GridAttackAnalyzer: A Cyber Attack Analysis Framework for Smart Grids

**DOI:** 10.3390/s22134795

**Published:** 2022-06-24

**Authors:** Tan Duy Le, Mengmeng Ge, Adnan Anwar, Seng W. Loke, Razvan Beuran, Robin Doss, Yasuo Tan

**Affiliations:** 1 School of Computer Science and Engineering, International University, Ho Chi Minh City 700000, Vietnam; ldtan@hcmiu.edu.vn; 2 Vietnam National University—Ho Chi Minh City, Ho Chi Minh City 700000, Vietnam; 3 School of Computing Technologies, RMIT University, Melbourne, VIC 3000, Australia; mengmeng.ge@rmit.edu.au; 4 Center for Cyber Security Research and Innovation (CSRI), School of Information Technology, Deakin University, Geelong, VIC 3216, Australia; seng.loke@deakin.edu.au (S.W.L.); robin.doss@deakin.edu.au (R.D.); 5 School of Information Science, Japan Advanced Institute of Science and Technology, Nomi 923-1211, Ishikawa, Japan; razvan@jaist.ac.jp (R.B.); ytan@jaist.ac.jp (Y.T.)

**Keywords:** smart grid, cybersecurity, cyber attack, vulnerability, graphical security modeling, attack graph, attack tree

## Abstract

The smart grid is one of the core technologies that enable sustainable economic and social developments. In recent years, various cyber attacks have targeted smart grid systems, which have led to severe, harmful consequences. It would be challenging to build a real smart grid system for cybersecurity experimentation and validation purposes. Hence, analytical techniques, with simulations, can be considered as a practical solution to make smart grid cybersecurity experimentation possible. This paper first provides a literature review on the current state-of-the-art in smart grid attack analysis. We then apply graphical security modeling techniques to design and implement a Cyber Attack Analysis Framework for Smart Grids, named GridAttackAnalyzer. A case study with various attack scenarios involving Internet of Things (IoT) devices is conducted to validate the proposed framework and demonstrate its use. The functionality and user evaluations of GridAttackAnalyzer are also carried out, and the evaluation results show that users have a satisfying experience with the usability of GridAttackAnalyzer. Our modular and extensible framework can serve multiple purposes for research, cybersecurity training, and security evaluation in smart grids.

## 1. Introduction

A smart grid refers to the inter-operation of electricity (and power) related technology, information technology, and communication technology, to improve the serving load of electrical power systems and facilitate the development of relevant end-user applications. In this next-generation electrical power system, the computing technology is integrated into the conventional electrical grid to improve different power network components’ connectivity, communication, and automation.

The Cabinet of Japan attempts to build new economic and social development strategies, “Society 5.0” [1], which debuted in 2016. The program envisions a future super-intelligent society that will benefit humanity with a better quality of life and a shift in social norms. The smart grid is described as one of the key concepts supporting this initiative. Furthermore, the U.S. Department of Homeland Security (DHS) [2] defined the smart grid as a “special” critical infrastructure supporting necessary services to sustain society and ensure economic development since it is essential to many of the 18 critical infrastructures.

Cybersecurity has become a major challenge for smart grid systems. For example, almost one-third of the cybersecurity incidents reported by the U.S. Industrial Control Systems Cyber Emergency Response Team (ICS-CERT) targeted the energy sector in 2014 [3]. Therefore, research on smart grid security needs to be enhanced. Due to its two essential parts, the power grid and network communication, the structure of the smart grid system is complex. In addition, the relationship between these two components needs to be considered for further research and improvement. Unfortunately, implementing a real smart grid system for cybersecurity experiments and validation is not trivial. Obviously, there is a considerable risk of damaging the electrical equipment and infrastructure, resulting in massive economic consequences or even putting human lives in jeopardy.

Fortunately, simulation testbeds and analytical techniques can be applied to conduct cybersecurity experimentation in the critical domains where testing on a real system is prohibited, such as smart grid. A smart grid simulation approach was applied in our related research in [4,5]. Besides, there is an increasing interest in the development of smart grid cyber-physical testbeds [6]. Along with their undeniable benefits, there are various disadvantages and drawbacks of these approaches, including costly and time-consuming implementation, scope limited to the current system, scalability problems, and mobility issues, as summarized in [7]. Consequently, the analytical approach is discussed and implemented in this research.

Through certain assumptions about how a method progresses, an analytical model is a mathematical abstraction that can be generalized to deal with different working conditions. In some instances, it is possible to determine a solution, and a result can be obtained in a wide variety of situations. The strength of an analytical model is that it provides a generalized method for obtaining performance results by using a mathematical formulation under different conditions. The model accuracy relies on ensuring the validity of the assumptions on which the mathematical formula is based. To estimate the modeling and measurement model, some uncertainties can be addressed with a stochastic model.

The application of analytical and simulation techniques for smart grid cybersecurity experimentation research has been increasing in recent years, from nearly 4000 papers in 2010 to more than 17,000 in 2019 (These figures were obtained from Google Scholar by using the search pattern as (“Smart Grid” OR “Smart Grids”) AND (“Analysis”) AND (“cybersecurity” OR “cyber security” OR “security”)). The attack analysis and simulation tools are principally utilized to investigate attacks and emulate their characteristics, especially network topologies and system settings. The use of real-world event simulation technologies for cybersecurity experimentation is believed to be the most critical aspect in improving the efficacy of the process. However, only a few research studies focus on the application of analytical techniques in conjunction with real security vulnerability data for the smart grid. To the best of our knowledge, this work is one of the first academic papers to examine this critical problem in-depth.

We start this article with a state-of-the-art literature survey of smart grid attack analysis. We then describe the design and implementation of GridAttackAnalyzer, a Cyber Attack Analysis Framework for Smart Grids, via graphical security modeling and security evaluations. To validate our framework, a case study involving a variety of attack scenarios is conducted. Using GridAttackAnalyzer, researchers can ascertain the repercussions of various attack types. In addition, our proposed framework facilitate the early development and evaluation of novel anomaly detection and mitigation techniques, even before they are implemented.

The main contributions of this paper are:

1. We applied graphical security modeling techniques and designed and implemented GridAttackAnalyzer, one of the first smart grid attack analysis frameworks, to enable researchers to easily create, modify the attack experimentation content, and facilitate their interaction with the system.
2. We conducted several case studies using various network models, power grid test feeders, and attack types to validate the proposed framework.
3. We performed a comprehensive comparison among the existing research on smart grid attack analysis and conducted an user evaluation for the GridAttackAnalyzer to demonstrate the usability of the framework.

The remainder of this paper is organized as follows. Background and related work are represented in Section 2. The architecture of GridAttackAnalyzer is explained in Section 3. In Section 4, the implementation and selected results of GridAttackAnalyzer are introduced. Section 5 discusses the functionality and user evaluations of GridAttackAnalyzer. Finally, Section 6 concludes the paper and points out future work directions.

## 2. Background and Related Work

A thorough overview of the most current analysis tools and their smart grid applications has been provided in our previous study [8].

Several approaches for modeling attackers’ behavior have been proposed recently. These approaches were designed to understand the system’s socio-technical perspective and explore how an intruder could influence the system’s functioning.

A smart grid system’s components, including IoT-enabled devices and essential communication protocols, are examined for security and safety risks in the work of Shama et al. [9]. In detail, the “multiarmed bandit” problem has been reformulated into an adaptive Bayes-based network security model. This new method examines how network defenders might efficiently distribute cyber defense teams among nodes, taking a dynamic approach to cyber security investment. The research finding can help answer the typical question of the smart grid system: How smart is smart enough?

M. Zekeriya et al. [10] provide an overview of cyber-attacks on power systems applications. In detail, this research introduces attacker types, attack types, and massive cyber-attacks on smart grid. In addition, a discussion of the most important goals and prerequisites for cyber security in smart grids is presented. In addition, the different kinds of attacks that can be carried out on smart grids are categorized according to the principles of confidentiality, integrity, and availability.

Liu et al. [11] contributed an i*-based framework, which is an agent-oriented requirement modeling language [12], for agent-oriented software engineering and requirement analysis. The work focuses principally on internal attackers rather than external hackers targeting a system. A similar approach was suggested by Mouratidis et al. [13] utilizing scenarios to demonstrate the nature of software security risks during the development process. A simple attacker model, where the attackers have to complete sub-goals to achieve the final target, was considered. Another study in [14] examined scenarios of attack during service layer application development. Obstacles were considered as the system’s anti-goals; hence, it is the attacker’s target. Asnar et al. [15] extended the i*-based framework by adding risks related to the system’s goal. However, the risk sources were not discussed in detail.

Beckers et al. [16] proposed a threat analysis structured method that involves the mapping of the attacker’s plan (documented in an attack tree) to particular system vulnerabilities (represented as an attack graph). They demonstrated that a part of an elaborate graph related to a particular target in the attack tree could be extracted. The research result showed that the complexity of the analysis of attack graphs was significantly reduced. Additionally, an algorithm to calculate an attacker’s overall success probability to reach the target was proposed in this research.

Ge et al. [17] proposed a framework for the security graphical modeling and evaluation of Internet of Things (IoT). The framework has five phases, including data processing, generation of security model, security visualization, security analysis, and model updates. An IoT Generator, a Security Model Generator, and a Security Evaluator were developed in this study. On the one hand, the IoT Generator’s role is to construct an IoT network based on the node vulnerability and network reachability data. In addition, based on the given IoT network, the Security Model Generator generates the extended Hierarchical Attack Representation Model (HARM). On the other hand, by using different security metrics, the Security Evaluator analyses the network’s security. The framework’s performance was evaluated by the implementation of the three distinct scenarios, including healthcare monitoring, smart home, and environmental sensing. The extended HARM [18] was applied to calculate all possible attack paths, and the selected security metrics values were determined in the security analysis process. The research encompasses a variety of security metrics, including Attack Graph Generation (AGG), Attack Tree (AT), attack cost (ac), attack impact (aim), attack success probability (*p*), and attack risk (*r*). In addition, the mathematical formulas for calculating security metrics were described in detail. The security decision-maker can identify the most vulnerable segment of the network from the study results, analyze the efficacy of various protection strategies, and decide the most efficient way to defend the network. Therefore, the impact of possible attacks can be mitigated. However, the framework addresses IoT systems issues in general, rather than focusing on smart grid attack scenarios specifically. Hence, there are still limitations in creating visualizations for attack graphs, likelihood, and application for smart grids.

Attack graph visualization is a useful approach for cybersecurity professionals and non-experts to investigate the system’s suspicious activities and examine all possible hacking attempts. The likelihood of an attack, which strongly enhances the risk evaluation process, can be defined using *p*. The lack of studies that focus on likelihood and attack graph visualization creates a gap in the smart grid field. Therefore, to bridge this gap, we employ the framework in this research.

## 3. GridAttackAnalyzer: Cyber Attack Analysis Framework for Smart Grids

**Figure 1:**
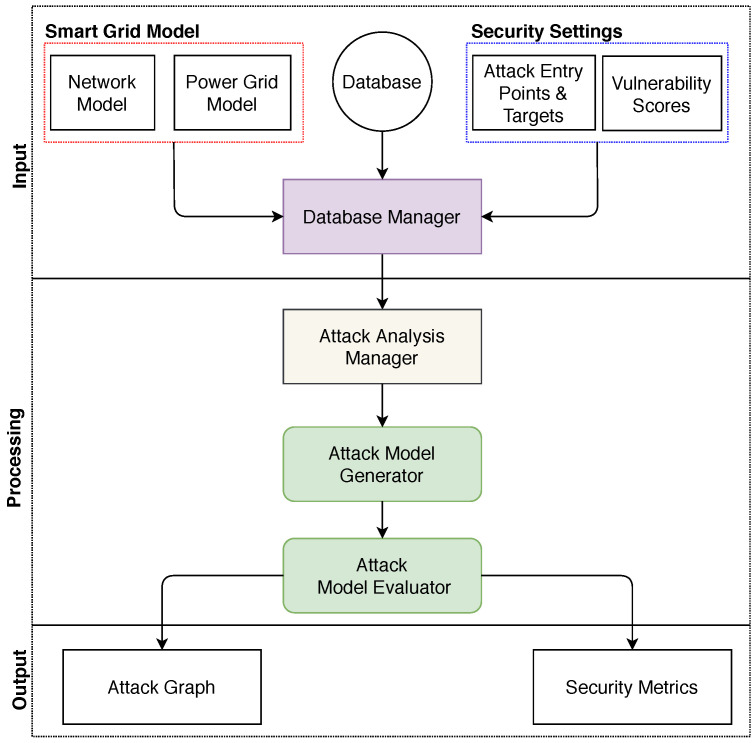
The architecture of GridAttackAnalyzer.

Based on an attack analysis approach where it is feasible to integrate the different smart grid components and provide the ability to analyze various cyber attack scenarios, we propose a Cyber Attack Analysis Framework for Smart Grids or GridAttackAnalyzer. Figure 1 illustrates the architecture of GridAttackAnalyzer. GridAttackAnalyzer is comprised of eight main components, including the database, smart grid model, security settings, database manager, attack analysis manager, attack model generator, attack model evaluator, and output (attack graph and security metrics).

### 3.1. Input

GridAttackAnalyzer investigates vulnerability exploitation attacks and evaluates their impact. By using the smart grid database, these attacks are instantiated. The database structure is provided in Table 1. It is organized by using a structured JSON-format file. Such a database is the input to enable reconfiguration to examine a wide variety of attacks on the same smart grid architecture. This searchable database comprises three sub-modules, including the smart grid model, smart grid devices, and Common Vulnerability Exposure (CVE) list [19], which is the publicly-known list of vulnerabilities and exposures.

The network model and power grid model are two critical parts of a smart grid system. To simulate each of these components, different studies have been conducted. On the one hand, various smart grid network architectural models were proposed in recent years [20]. These network models were formed by the connections between smart grid devices. Since GridAttackAnalyzer aims to allow the users to optimize the network security, these connections are not physically stored in the database. The network model is configured by users later. On the other hand, various distribution test feeders, which vary in control data, complexity, and scale have been developed [21]. Each test feeder contains several residential loads or houses organized into smaller areas to facilitate attack analysis. This information is stored in the database.

A smart grid involves various energy measures and operations; for instance, smart meters, smart appliances, and Supervisory Control and Data Acquisition (SCADA). A smart meter or a smart electric energy meter is a piece of equipment that measures electrical data (e.g., current, electricity consumption, power factor, and voltage levels). Smart meters enhance the visibility of energy usage, power consumption behavior, and customer billing. Further, they enable various smart grid applications, for instance, dynamic pricing and demand response. Smart appliances have the ability to respond to the dynamic pricing and demand response signals. These applications add additional value for smart grid appliances through intelligent control, power management, and network technologies. In addition, one feature of designing the capability of a smart grid is incorporating SCADA systems to allow the utilities to track and control network equipment remotely. The information gathered by these smart meters, smart appliances, and SCADA devices are organized in the database. The structure includes ID, name of the device, the CVE list of the device, group, and description.

### 3.2. Processing

Each of the smart grid devices has a corresponding CVE List. The list is collected from the National Vulnerability Database (NVD) website [22] by searching the name of a smart grid device. Each CVE is stored in the database under the components of the CVE List sub-module.

The database manager module is the interface that interacts with end-users, attack analysis manager, and the database to capture and analyze an attack. It first obtains data from the database, then enables the users to select the power grid and network model from the smart grid model module, attack entry point, attack target, and vulnerability scores from the security settings module. The information is then transmitted to the attack analysis manager module to start the processing stage.

#### 3.2.1. Attack Analysis Manager

The attack analysis manager serves as the engine of GridAttackAnalyzer. It initializes the running environment, and the configuration of the network model, the power grid topology, and the security setting. Additionally, this attack analysis manager controls the composition of the attack analysis scenarios and manages the attack model generator and attack model evaluator.

When analyzing a scenario, the attack analysis manager module uses the data from the data manager module to prepare the analysis environment. Then, the data is transferred to the attack model generator for the next steps.

#### 3.2.2. Calculation of Security Metrics

The security metrics are calculated using the security model generator and security model evaluator modules proposed in [17]. When the network is constructed, the security model generator module takes the network topology and vulnerability information as inputs to compute all possible attack paths in the smart grid network. For reference, a summary of mathematical symbols appearing throughout is given in Table 2.

Vulnerability scanners are widely used to identify a network’s security vulnerabilities and their components, including type, number, and location. The CVE employs the Common Vulnerability Score System (CVSS) [23] to determine the severity levels of these vulnerabilities. For the attack analysis process, these CVSS scores can be utilized as inputs to generate the Graphical Security Model (GrSM) [24]. This model depicts how a system can be compromised via various attack vectors. Therefore, solutions to defend against these threats can be formulated. The two essential parts of GrSM are the Attack Graph (AG) [25] and Attack Tree (AT) [26]. ATs are conceptual diagrams that represent the various ways in which an asset or target can be compromised. An AG is a concise representation of all paths through a system that ends with an attacker achieving their target successfully. The advantages of the AT and AG application for smart grid attack analysis are discussed in our related research in [8].

In this approach, we denote a node as *t*, a set of nodes as *T* where t∈T, a vulnerability as *v*, a set of vulnerabilities for node *t* as $V_{t}$. Vulnerabilities in each node are constructed as an AT (vulnerabilities as leaf nodes; AND gate if all vulnerabilities need to compromised to gain the privilege; OR gate if only one of the vulnerabilities needs to be compromised to gain the privilege) via the graphical security model, denoted as $at_{t}$. We define an attack path, ap is a sequence of nodes that can be compromised by an attacker along the path from an entry point to an attack target in the AG. Each AG has a set of attack paths AP (from each possible entry point to each possible attack target) where ap∈AP. We define security metrics in node-level, path-level, and network-level and describe the calculation of these metrics.

The vulnerability’s attack success probability (*p*) is the value used to estimate the likelihood of an attacker succeeding in exploiting the vulnerability. At the node level, the metric is used to measure the probability of success when an attacker compromises the node. We first calculate attack success probability for each inner node *g* in $at_{t}$ where g∈{AND,OR}, denoted as $p_{g}$ and measured by Equation (1); we then calculate $p_{t}$, which is the root value in $at_{t}$.

(1) $$p_{g}=\{\begin{array}{c} \Pi_{v\;\in \;V_{g}}p_{v};g=AND\\ 1-\Pi_{v\;\in \;V_{g}}\left(1-p_{v}\right);g=OR \end{array}$$

where $p_{v}$ is the attack success probability value of a vulnerability and $V_{g}$ is the set of vulnerabilities under gate *g*. At the path level, the value of attack success probability is measured by Equation (2). This value represents the hacker’s ability to access a target through a specific attack path.

(2) $$p_{ap}=\prod_{t\in ap}p_{t};\;ap\in AP$$

At the network level, the metric value is the maximum attack success probability of the path among all possible attack paths.

(3) $$P=\max_{ap\in AP}p_{ap}$$

The quantifying value of the cost that an attacker spend for exploiting a vulnerability is called an attack cost (ac). At the node level, the metric is used to measure the attack cost for successfully compromising a node. For node t∈T of an attack tree and each inner node, the value of attack cost for a node is calculated by Equation (4). At the path level, the measure is the cost spent by an attacker to compromise the target over the attack path. This cost is calculated by Equation (5).

At the network level, the measure is the minimum attack cost to compromise the target along one attack path among all possible paths. The cost of network level is given by Equation (6).

(4) $$ac_{g}=\{\begin{array}{c} \sum_{v\;\in \;V_{g}}ac_{v};g=AND\\ \min_{v\;\in \;V_{g}}ac_{v};g=OR \end{array}$$

(5) $$ac_{ap}=\sum_{t\in ap}ac_{t};\;ap\in AP$$

(6) $$AC=\min_{ap\in AP}ac_{ap}$$

The attack impact (aim) of a vulnerability is a quantitative representation of the possible damage that an attacker could cause by exploiting a vulnerability. At the node level, the metric is used to measure the potential damage that an attacker could cause by compromising a node. The value of attack impact for a node is calculated by Equation (7). The aim value of node *t* is defined as the aim of the root node.

Similarly, as indicated in Equation (8), the aim value of an attack path is calculated by adding the attack values of each node *t*. Then, a network-level attack impact is a maximum impact of one attack path among all possible paths taken, as shown in (9). The aim values are calculated by the following formulas:

(7) $$aim_{g}=\{\begin{array}{c} \sum_{v\;\in \;V_{g}}aim_{v};g=AND\\ \max_{v\;\in \;V_{g}}aim_{v};g=OR \end{array}$$

(8) $$aim_{ap}=\sum_{t\in ap}aim_{t};\;ap\in AP$$

(9) $$AIM=\max_{ap\in AP}aim_{ap}$$

The risk of a vulnerability (*r*) is the probability of loss resulting from the vulnerability exploitation. At the node level, the metric is the probability of loss from the node compromise. It is computed by summing the product of the probability of attack success $pr_{a}$ and the amount of damage $aim_{a}$ on an attack path ap, as in Equation (10). Similarly, at the path level, *r* is calculated by $pr_{t}$ and $aim_{t}$ values of node as shown in Equation (11). The *R* value of the network is the maximum value of $r_{ap}$, as explained in Equation (12).

(10) $$r_{g}=\{\begin{array}{c} \sum_{v\;\in \;V_{g}}pr_{v}\times aim_{v};g=AND\\ \max_{v\;\in \;V_{g}}pr_{v}\times aim_{v};g=OR \end{array}$$

(11) $$r_{ap}=\sum_{t\in ap}pr_{t}\times aim_{t};\;ap\in AP$$

(12) $$R=\max_{ap\in AP}r_{ap}$$

By using the security metrics, the security evaluator can perform three functions. The first is to produce the analysis results directly. The second is to generate and export a CSV-format output file. The final function is to generate the AG automatically. In addition, attack paths are classified based on the five-level of attack success probability, ranging from almost certain, likely, possible, unlikely, and rare.

### 3.3. Output

Data output is an essential part of any analysis system, and our attack analysis on the smart grid is no exception. Attack Graph and various security metrics, including attack cost, attack success probability, attack risk, attack impact, and likelihood, are the outputs of GridAttackAnalyzer. After finishing the attack analysis process, the output in CSV (comma-separated values) format can be loaded. It is a simple file format used mainly to store tabular data; for instance, a spreadsheet or a database. By using the user-friendly GUI, the analytical outputs can be selected and visualized. GridAttackAnalyzer allows users to generate an AG automatically. Furthermore, attack paths are classified by likelihood based on the probability ranges. This function facilitates the users in making a qualitative comparison between the attack scenarios quickly. Consequently, the characteristics of the attacks can be easily distinguished. Currently, bar graphs are supported.

## 4. Implementation and Analysis Results of GridAttackAnalyzer

**Figure 2:**
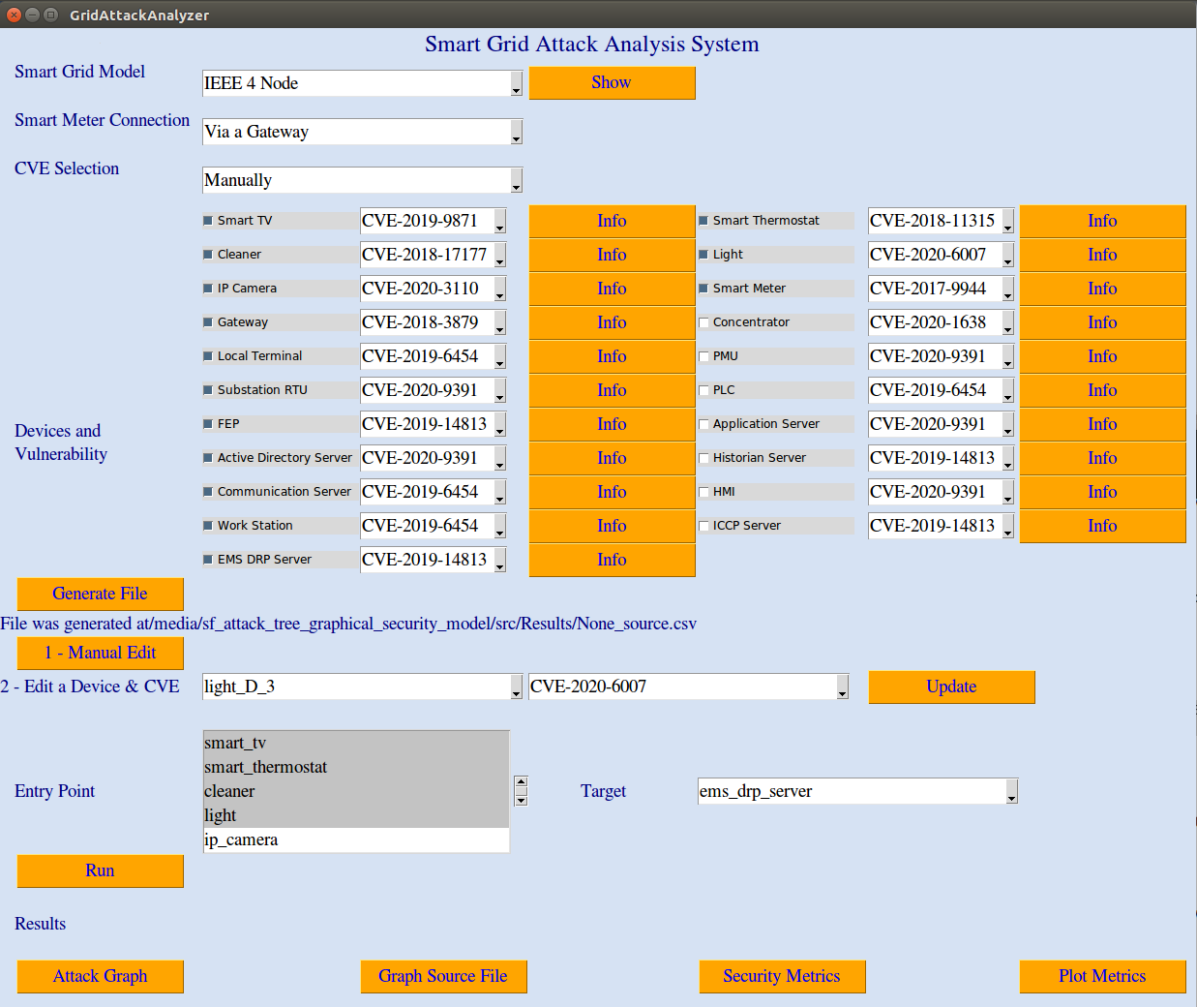
GridAttackAnalyzer desktop application.

We discuss the proof-of-concept prototype of the proposed framework in this section. Using a Python binding to the Tk GUI toolkit named Tkinter [27], we implemented the framework as a smart grid attack analysis desktop application. The user interface of GridAttackAnalyzer is depicted in Figure 2.

### 4.1. Smart Grid Model

In the smart grid research community, the Pacific Northwest National Laboratory (PNNL) taxonomy feeders [28] and IEEE feeders [29] are commonly applied from the power grid simulation research. For the network model, there have been numerous network architecture designs for the smart grid system [30,31]. We have employed the IEEE feeders in our previous research in [4,5]. Hence, in the scope of this study, PNNL taxonomy feeders will be used. Note that GridAttackAnalyzer is designed to integrate more network and power grid models. Therefore, the models applied in the case studies can not be considered as the only ones.

#### 4.1.1. Power Grid Model

We consider the following scenario. The expanding incorporation of smart grid technologies in the U.S. power network demonstrates the value of test feeders’ availability, enabling the effect of attacks on cyber-physical models to be assessed.

The current US power grids have a wide variety of topologies and appliances due to their vast scale and numerous services. Hence, test feeders should also represent these variations based on factors such as voltage and climatic area. In 2009, PNNL developed a 24-node taxonomy radial distribution test feeder reflecting the U.S. continental region. Through a clustering algorithm involving 17 different utilities and their 575 current test feeders, these distribution test feeders have been created. In order to implement this categorization, the mainland region was split into five climate zones, where 35 statistical and electrical properties were studied.

**Figure 3:**
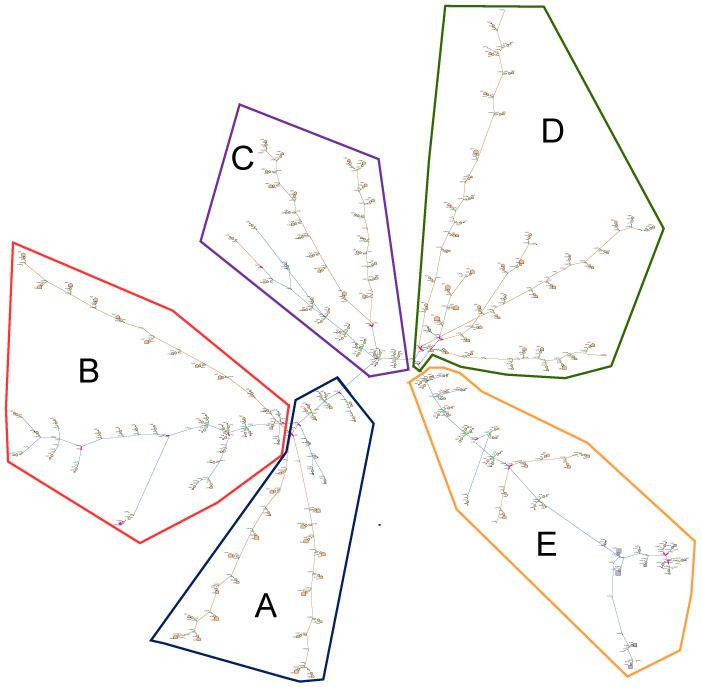
R4-12.47-2—A PNNL taxonomy feeder [32], where A, B, C, D, and E, are the corresponding areas.

R4-12.47-2 gains its advantage from the 24 prototypical feeders by combining a moderately populated urban area with a small suburban area. In addition, there are mainly one-family homes in the less populated city, which is appropriate for our case study. Figure 3 shows the R4-12.47-2 infrastructure which consists of 352 houses. A smart meter was attached to each house to collect electric energy consumption data. These houses are divided into five smaller areas, A, B, C, D, and E, to improve the control of performance.

#### 4.1.2. Network Model

The smart grid in this study consists of three interdependent networks including Wide Area Network (WAN), Neighbor Area Network (NAN), and Home Area Network (HAN) [33]. To reflect network relationships with the utility, the research [34] proposed two different types of HAN architectures. The smart meter controls all house devices in the first architecture. This design’s drawback is that all smart home appliances have to exchange data using the same communication technology. In order to handle the complexity of several network n protocols, the second architecture, in which all appliances are connected to the smart meter via a gateway, was designed.

**Figure 4:**
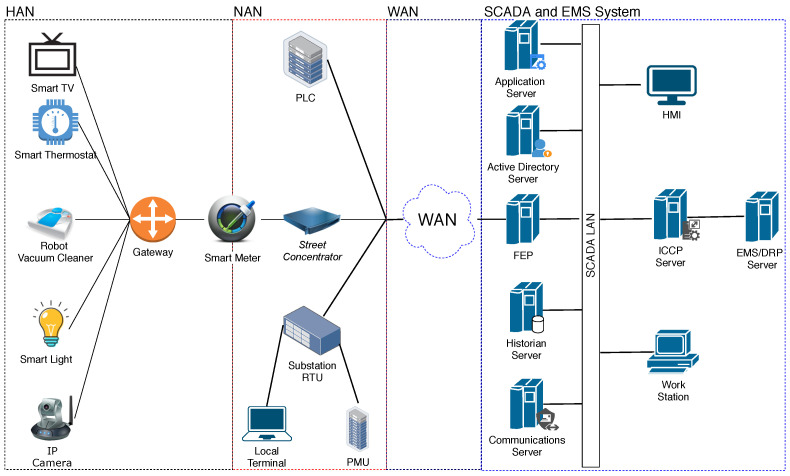
An example of the smart grid simplified network model with a gateway.

Based on the power grid’s selected configuration, the smart grid communication network with the gateway is illustrated in Figure 4. For the sake of our case study, we note that this network model has been simplified. Each residential house in the network model represents a residence in the power grid model. Furthermore, in the electricity grid model, these residential houses are clustered into smaller areas in the same way that individuals are aggregated. Smart appliances such as an IP camera, smart TV, smart light, smart thermostat, and smart vacuum, are installed in every home. Through using the gateway, smart appliances transmit the collected data to the smart meter. Then, the data was forwarded to the associated street concentrator. Finally, data is obtained by the SCADA system.

A Front End Processor (FEP) is a computing device that interfaces to the SCADA system. For practical reasons, such as avoiding the need for a new pair of modems, FEP can be considered a central node in the network model. Its function is to establish a solid communication link from HAN and NAN devices, for instance, the street concentrators and substations. Furthermore, it ensures the connection with the SCADA system. FEP aims to offload the SCADA system from transmitting and receiving data, managing the peripheral devices, error correction and error detection, and packet assembly and disassembly.

Since the power system goes through numerous operating states such as normal, alert, emergency, and restorative, Energy Management Systems (EMS) is designed to maintain the capability of the system by monitoring its behavior and making decisions to get it back to normal operation. EMS also supports the demand response (DRP) application. The operation of EMS relies on data acquired by SCADA. It is at the top level of our applied network model. Due to the scope of this research, other devices are not discussed in detail.

An ID is assigned to each smart appliance or node in the system following a predetermined pattern, comprising the device name, area, and home ID. For example, the ID of an IP camera belongs to area A’s house number 2 is denoted as $Cam_{A_{2}}$. Similarly, $Thermostat_{B_{2}}$, $Cleaner_{B_{2}}$, $Light_{B_{2}}$, $TV_{B_{2}}$, $Gateway_{B_{2}}$, and $Meter_{B_{2}}$ represent the devices in the second house of the area B. In addition, $Concentrator_{A}$, $Concentrator_{B}$, $Concentrator_{C}$, $Concentrator_{D}$, and $Concentrator_{E}$ are the concentrator IDs for each area A, B, C, D, and E, respectively. Local_Terminal, RTU, FEP, Communication_Server, ICCP, and EMS/DRP serve as the IDs for the local terminal, substation RTU, FEP, Communications, ICCP, and EMS/DRP servers in our simplified network model (part of smart grid).

### 4.2. Devices and Vulnerabilities

Vulnerabilities are serious security flaws that hackers can exploit to compromise a susceptible network. Attackers can use sequences of software, open-source exploit kits, or commands to explore security vulnerabilities and carry out malicious activities. This research assumes that attackers can exploit the vulnerabilities listed in Table 3. Any HAN device, consisting of smart thermostats, smart TVs, robot vacuum cleaners, IP cameras, and smart lights, one by one or even all of them, can be used as the entry points to start an attack. Further, some smart grid devices in the substations and the SCADA system can be used as the attack’s entry points.

### 4.3. Attack Scenarios

According to the report by the European Union Agency for Cybersecurity (ENISA) [35], channel jamming, DNS attacks, injection attacks, and malicious code are among the attacks that should be considered in Smart Grid area. The simulation of these attack types are detailed in our related research in [4]. In this work, we focus on vulnerability exploitation attacks. Once attackers control the devices via vulnerability exploitation attacks, they can launch further attacks (e.g., denial of service attacks, injection of false information).

This research assumed all that smart appliances in seven households, which are nearly 2% of the system’s 352 residential houses, have vulnerabilities. In particular, one house in areas C, D, and E and two households in areas A and B contain vulnerabilities.

We perform five case studies in the context of scenarios for training learners/trainees about cybersecurity attacks on smart grids via IoT devices. Four case studies consider entry points in HAN because IoT devices can be easily compromised by attackers and used as stepping stones to reach other attack targets; the last case study considers the local terminals for substations as entry points in the NAN to show that these devices can be compromised and used as entry points to break into the SCADA system.

1. Multiple-entry single-target attack model (entry devices of single type): we assume one type of device has vulnerabilities in this case study. Consequently, in order to launch an attack, attackers can only manipulate this type of device within the infected houses. For example, all IP cameras in the seven selected residential houses contain different types of vulnerabilities. Therefore, these infected IP cameras can be exploited as attack entry points. This basic scenario is used to introduce the trainees to the system’s functions.
2. Multiple-entry single-target attack model (entry devices of multiple types): vulnerabilities exist in all types of devices in the seven selected houses. Therefore, attackers would probably manipulate any of the equipment in order to execute an attack. This circumstance aims to equip the users with attack analysis ability.
3. Multiple-entry single-target attack model with patch: patching refers to the process by which the vulnerabilities in a specific device is repaired. The case study extends the multiple-entry single-target attack model under multiple device types scenario to incorporate patches’ deployment as a defense strategy. For instance, all smart TVs’ security vulnerabilities have been resolved. Since the issues are fixed, they are not appropriate entry points for the attacker to start an attack. This training scenario is applied to introduce the trainees to the patching functions and evaluate the effectiveness of a given defense strategy.
4. Multiple-entry multiple-target attack model: this case is the extension of the multiple-entry single-target attacker model under multiple device types by expanding the attack target to the SCADA system’s core. This scenario aims to demonstrate the massive attack analysis ability of the training system. The users can learn how a large-scale attack happens and what the consequences are.
5. Attack model with local terminals for substations as entry points: all local terminals of the substations have vulnerabilities. Hence, attackers would probably manipulate any of these local terminals as the entry points to conduct an attack. This case study aims to show how a physical device controlled by the SCADA system might be hacked and leads to the compromise of the SCADA system.

The attack targets in these scenarios are the SCADA system’s devices. In detail, the case studies from 1 to 3 aim to control the FEP, while EMS/DRP is targeted in the 4th and last scenarios. If a smart grid device has multiple vulnerabilities, attackers can choose one at random to use in their attempt.

The GridAttackAnalyzer enables users to construct a network model by selecting smart grid devices, as well as an attacker model by choosing potential entry points and attack targets. Therefore, the considered attack scenarios in this paper are illustrative. The framework allows trainers to create new network models based on chosen devices, assign vulnerabilities, and modify CVE values easily. Hence, more attack scenarios can be analyzed.

### 4.4. Attack Analysis Execution and Result Visualization

**Figure 5:**
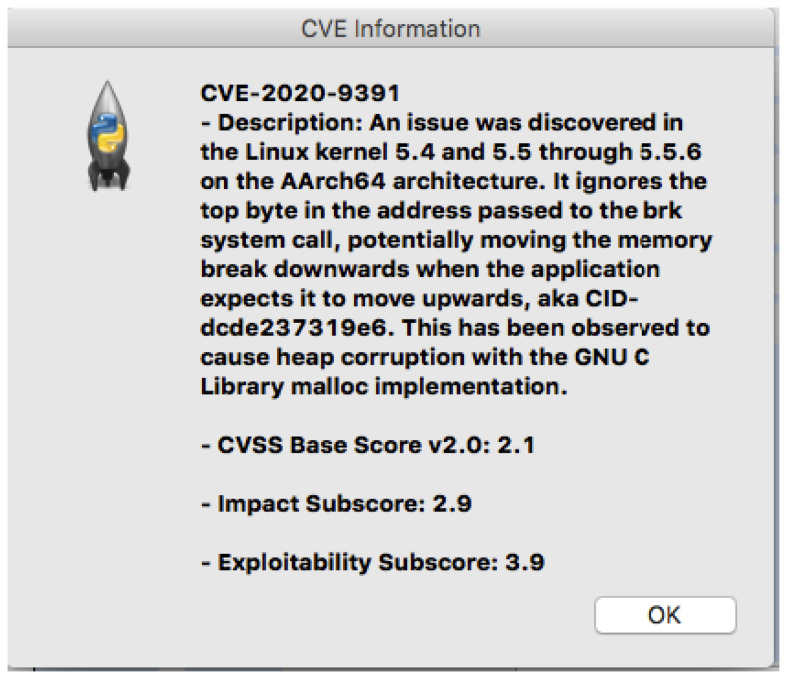
An example of CVE information.

To start an attack analysis session, a trainee (or the system’s user) selects a smart grid model. There is a “Show” button next to the smart grid model dropbox to visualize the smart grid model structure. Next, smart grid connection and CVE selection types should be selected. Currently, two smart meter connection types, including “via a gateway” and “direct connection”, as well as two CVE selection types, namely, “manually” and “automatically”, are supported. Devices and the corresponding vulnerability should be selected by clicking on their checkboxes. An “Info” button is located next to a corresponding CVE to show the CVE information, including CVE description, CVSS Base Score v2.0, Impact Subscore, and Exploitability Subscore. An example of CVE information is shown in Figure 5.

After the smart grid model, smart meter connection, CVE selection type, and devices and vulnerability have been chosen, the system is ready to create the source file by the user clicking on the “Generate File” button. This source file is a CSV-format file that contains all of the necessary data for an attack analysis session. GridAttackAnalyzer enables users to modify this source file before starting an analysis session by two options. The first option is to open the CSV-format file and manually change the data. This option allows the trainees to modify the source file freely. However, it is sometimes tiresome and error-prone. Another option is to select a specific IoT device and update its CVE information. By using this option, the error-prone issue can be eliminated.

When the source file is ready and the entry points and targets are selected, the attack analysis session is ready to start by clicking on the “Run” button. After finishing the attack analysis process, the outputs are stored in the CSV-format files. The attack graph source file, which contains the information of all attack paths, can be accessed by clicking on the “Graph Source File” button. All paths can be gathered to form an attack graph and visualized by clicking the “Attack Graph” button. On the other hand, the calculated security metrics are also archived in a CSV-format file. It can be accessed by selecting the “Security Metrics” button. Finally, these security metrics can be visualized for the result comparison among different attack scenarios. Currently, the bar chart option is supported.

### 4.5. Analysis Results

By applying the mathematical formulas discussed in Section 3.2.2, the security metrics values, including attack cost (ac), attack success probability (*p*), attack impact (aim), and attack risk (*r*), are calculated in node, attack path, and network levels. Attack paths are classified into five categories based on the range of *p* taken from [36,37], including almost certain (0.8 ≤ *p* ≤ 1), likely (0.6 ≤ *p* ≤ 0.79), possible (0.4 ≤ *p* ≤ 0.59), unlikely (0.2 ≤ *p* ≤ 0.39), and rare (0.0 ≤ *p* ≤ 0.19) paths. These categories are summarized in Table 4. In addition, Table 5 shows the network-level analysis results. In detail, the first five scenarios represent the results for the multiple-entry attack model under one device type, the sixth scenario shows the results for the multiple-entry attack model under multiple device types, the seventh and eighth scenarios demonstrate the results for the multiple-entry attack model with patch, the ninth scenario represents the large-scale attack on the smart grid system, and the last one shows the result of the substation to SCADA system scenario.

#### 4.5.1. Multiple-Entry Single-Target Attack Model under One Device Type

It is clear that compromising the smart lights and smart TVs incurs the highest success probability (*p*) of 1. However, the attack cost (ac) associated with attacking the smart lights is higher than those targeting smart TVs. As a result, attackers can access the FEP via 16 smart TVs’ entry point paths, eight of which are almost certain. Consequently, hackers are more motivated to target smart TVs as entry points (if they are aware of the vulnerabilities).

Attack cost at the network level refers to the most negligible possible cost, whereas attack impact refers to the most significant loss caused by an attacker attempting to compromise the target via all possible paths. As a result, even in the single-entry attacker model, an optimal path for attackers to compromise the victim may not exist. For example, the following path from $TV_{A_{2}}$ to FEP has the maximum attack risk (*r*) and impact (aim) at 33.9, maximum attack success probability at 1, and minimum attack cost at 21.7:

- Attackers →$TV_{A_{2}}$→$Gateway_{A_{2}}$→$Meter_{A_{2}}$→$Concentrator_{A}$→FEP

However, the following path from $TV_{B_{1}}$ to FEP has the maximum impact at 33.9 but lower attack success probability:

- Attackers →$TV_{B_{1}}$→$Gateway_{B_{1}}$→$Meter_{B_{1}}$→$Concentrator_{B}$→FEP

Once the smart grid system has been analyzed, an attacker can decide which paths to compromise in order to achieve their goal. Security specialists can use this knowledge to defend the system against an attack.

#### 4.5.2. Multiple-Entry Single-Target Attack Model under Multiple Device Types

By increasing the number of entry points, attackers gain additional attack vectors. It is highly likely that the smart grid system will be attacked, as there are 17 almost certain, 20 likely, and 14 possible paths among 80 potential ones. In this case, hackers need to spend less cost at 19.7. Unfortunately, the attack risk and attack impact are at their maximum of 33.9. Therefore, to prevent intruders from hacking into the system, smart lights and smart TVs should also be secured first.

#### 4.5.3. Multiple-Entry Single-Target Attack Model with Patch

We independently change the vulnerability data for (1) both smart TVs and smart lights or (2) smart TVs only.

Due to the fact that the potential attack vectors are created by both smart lights and smart TVs, the effect of patching for smart TVs is not immediately apparent. The attack risk, attack impact, and attack success probability, remain the same as in the previous model. In addition, the total number of paths has been reduced. The number of paths with almost certain likelihood is reduced to 9.

By mitigating vulnerabilities in smart lights and smart TVs, we reduce the likelihood of an attack success and the associated risk. Unfortunately, the attack impact and attack cost remain unchanged. This is because of the smart thermostats and IP cameras, which cost attackers less effort to compromise but can cause more significant consequences. There are 5 almost certain paths. As a result of the findings, it is clear that securing both smart lights and smart TVs is more beneficial than defending each of them separately.

#### 4.5.4. Multiple-Entry Multiple-Target Attack Model

In this case study, attackers can use all of the HAN devices to start an attack. The target is the EMS/DRP server. Since more entry devices are provided, there are more paths to conduct an attack. There are a few serious vulnerabilities in this scenario. Therefore, the attack success probability is just 0.33, and the attack risk is just 14.15. Among 125 attack paths, there are 59 unlikely paths and 66 rare paths. However, attackers need to spend more effort since they have to compromise more devices to reach the target. The attack cost is 22.09, which is the highest in the scenarios. Similarly, the attack impact is high at 36.29. Therefore, more effort is required to conduct this attack. However, there is an enormous consequence if attackers achieve the target. The following is an example of an attack path which has the highest attack impact:

- Attackers →$TV_{C_{2}}$→$Gateway_{C_{2}}$→$Meter_{C_{2}}$→$Concentrator_{B}$→FEP→ICCP→EMS/DRP

#### 4.5.5. Attack Model with Local Terminals for Substations as Entry Points

In this scenario, the substations’ local terminals were employed as the entry points for the hackers to conduct an attack. EMS/DRP server is also the target of this case study. Since the CVSS values of the local terminals and substation RTUs are not high, the maximum attack success probability is low as p=0.05. There are 12 paths that allow attackers to compromise the EMS/DRP server. Fortunately, all of them are rare paths. However, the attack cost for an attacker to achieve their goal is still high as c=18.57, and the attack impact is 22.17. Therefore, severe damage can be still observed if the EMS/DRP server is compromised. Due to the low attack success probability, the attack risk is low at 1.131.

The path from $Local\_Terminal_{B}$ to EMS/DRP, which is shown in the following, has the maximum attack success probability:

- Attackers →$Local\_Terminal_{B}$→$RTU_{B}$→FEP→ICCP→EMS/DRP

#### 4.5.6. Example of Result Visualization

**Figure 6:**
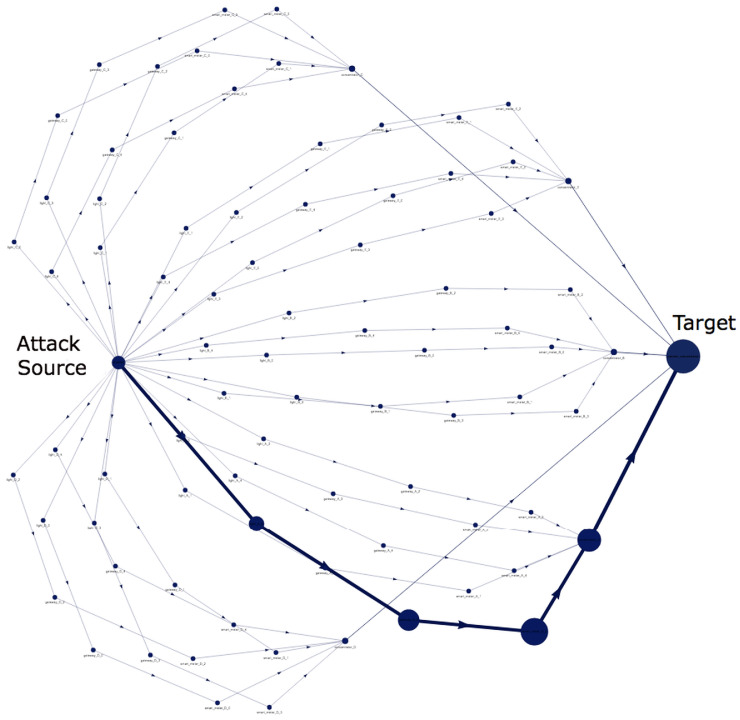
An illustration of an attack graph produced as part of a case study.

One of the main functions of GridAttackAnalyzer is to analyze the attacks on the smart grid system. To enable users to understand the attack graphs easily, GridAttackAnalyzer automatically generates the attack graphs. The visualization shows all of the possible attack paths for attackers to reach the targets. An attack graph that needs to be considered, for instance, with the highest value of the selected metric, is highlighted in the graph. The automatic attack graph generation is one of our contributions to fill the gap in current work. For instance, by utilizing attack success probability, an attack graph with all possible attack paths is automatically constructed by GridAttackAnalyzer from scenario Section 4.5.1 as shown in Figure 6. The highlighted path is from $TV_{A_{2}}$ to FEP, which has maximum attack success probability at 1:

- Attackers →$TV_{A_{2}}$→$Gateway_{A_{2}}$→$Meter_{A_{2}}$→$Concentrator_{A}$→FEP

Along with the CSV format output files, GridAttackAnalyzer allows users to visualize the results. The security metrics, including attack impact, attack success probability, attack risk, and attack cost, can be visualized. Furthermore, the number of attack paths classified as almost certain, likely, possible, unlikely, and rare can be visualized in charts.

**Figure 7:**
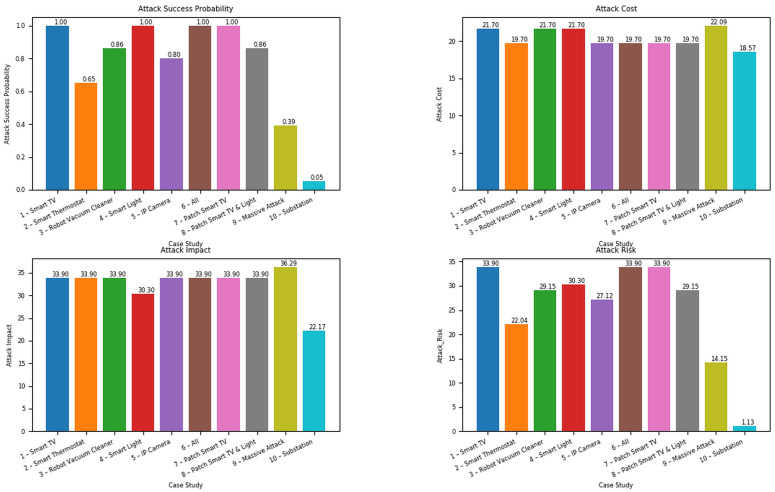
An example of attack analysis result visualization.

The data in CSV-format output files are too numerous or complex to be represented appropriately here, due to space limitations. However, we note that this function enables the trainee to compare the results of different attack scenarios. Using charts, data can be displayed, and further exploration of an analysis result can be facilitated. An example of a visualization of attack analysis results is shown in Figure 7. Currently, the bar chart type is supported.

## 5. Evaluation

We report on an evaluation of our system based on two aspects: a comprehensive functionality comparison and scenario analysis by selected users.

### 5.1. Functionality Comparison

#### 5.1.1. Evaluation Method

Our related research indicated that a useful smart grid experimentation system should integrate both the network and power grid models with security components [38]. Therefore, GridAttackAnalyzer meets the requirements for smart grid cybersecurity experimentation with the combination of these components. To highlight the useful functions of GridAttackAnalyzer, we compare its functionalities with related research. More specifically, GridAttackAnalyzer is evaluated by comparing the ability to calculate various interest metrics, including likelihood, attack cost, attack success probability, attack risk, and attack impact.

#### 5.1.2. Evaluation Results

For smart grid attack analysis, most research only considers limited attack metrics calculations when hackers attempt to compromise the cyber-physical system. Furthermore, attack graph visualization and likelihood are also typically not included in those implementations, unlike our work. Additionally, smart grid attack analysis is still a new area of research. To the best of our knowledge, GridAttackAnalyzer is one of the pioneering frameworks for smart grid attack analysis. Hence, GridAttackAnalyzer is more comprehensive than other related frameworks in terms of smart grid application and security metrics calculations, including a wide range of metrics, i.e., attack cost, attack risk, attack success probability, attack impact, as well as likelihood, as shown in Table 6.

### 5.2. User Evaluation

#### 5.2.1. Evaluation Method

We also conducted an external user evaluation. Particularly, ten participants, who are Ph.D. candidates in cybersecurity or related topics, were invited to use GridAttackAnalyzer. There were five participants from JAIST and five from other institutions.

We had a session to introduce the functions of GridAttackAnalyzer to each participant. After this session, a user guide was provided to the participants. Each of the 10 Ph.D. students attempted to carry out the case studies introduced in Section 4. All the participants succeeded in reproducing the case studies results. Moreover, they were encouraged to use the frameworks to simulate and analyze new case studies, for example, to change attack entry points or targets with the new CVE values. After completing the experiment, all participants were asked to complete a usability questionnaire to measure their satisfaction with the frameworks’ cognitive-load.

A reliable tool for measuring the usability, the System Usability Scale (SUS), was applied to enable users to respond to a usability questionnaire. This well-known standardized questionnaire was first introduced in 1996 by Brooke [39] and is accounted for more than 40% of post-test questionnaire usage [40]. The structure of SUS is simple with a ten-item attitude Likert scale, ranging from 1 for “strongly disagree” to 5 for “strongly agree”. Even for a small sample of participants, it has been proven to produce highly reliable results [41]. The outcome of SUS is a single score on a scale from 0 to 100. The qualitative interpretation of SUS scores is defined in [42] as follows:

- 0 ≤ SUS Score < 36: Poor
- 36 ≤ SUS Score < 51: OK
- 51 ≤ SUS Score < 72: Acceptable
- 72 ≤ SUS Score < 85: Good
- SUS Score ≥ 85: Excellent

**Figure 8:**
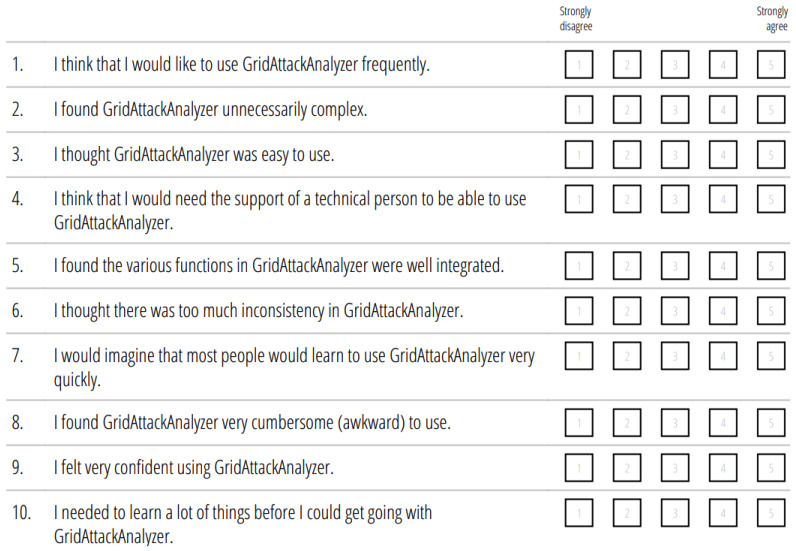
SUS questionnaire for GridAttackAnalyzer.

The questions used in the questionnaire for GridAttackAnalyzer are shown in Figure 8. Among the 10 questions, five are positive and five are negative, and negative items alternate with positive ones. By listing these negative and positive statements in an alternating way, the participant is challenged to to read every question and try to think whether they agree with it or not (other possible randomized interleaving of positive and negative questions can also be used).

The score contributions from each question, ranging from 1 to 5, were used to calculate the SUS score by Equations (13)–(15), and were explained as follows:

- The score contributions from the odd items: the scale position minus 1.
- The score contributions from the even items: 5 minus the scale position.
- The overall SUS value in the range of 0 to 100: the 10 question’s total score is multiplied by 2.5.
- The mean SUS score is the average SUS scores of *n* participants.
  (13) $${\mathrm{Score}}_{i}=\{\begin{array}{c} a_{i}-1,\;\mathrm{if}\;i\%2\neq 0\\ 5-a_{i},\;\mathrm{if}\;i\%2=0 \end{array}$$
  (14) $${\mathrm{SUS}}_{j}=2.5\times \sum_{i=1}^{10}{\mathrm{Score}}_{i}$$
  (15) $$\bar{\mathrm{SUS}}=\frac{\sum_{j=1}^{n}{\mathrm{SUS}}_{j}}{n},n\in N$$

#### 5.2.2. Evaluation Results

The analysis reflects the result values of SUS for GridAttackAnalyzer, which is shown in Table 7. Standard deviation, which is the dispersion measure of a data set from its average, was calculated by the (16) equation where σ is the data standard deviation, *N* is the data set size, $x_{i}$ is defined for each value, and μ is the mean of the data set.

(16) $$\sigma =\sqrt{\frac{\sum {\left(x_{i}-\mu \right)}^{2}}{N}}$$

The SUS mean score is 72.2 for GridAttackAnalyzer. These mean scores can be considered as good ($\bar{SUS}>72$) for the framework. The standard deviation is 10.2 for GridAttackAnalyzer. Furthermore, the minimum scores are above 60, which is acceptable. Comparing these usability values, we can see that the users had satisfying experiences with the framework’s usability, generally speaking. Although almost all the participants agreed that most researchers would learn to use GridAttackAnalyzer very quickly, two out of ten found GridAttackAnalyzer unnecessarily complex. Therefore, we should consider this aspect carefully for future development.

## 6. Conclusions

In this paper, we introduced GridAttackAnalyzer, a framework for cyber attack analysis on smart grids. Its user-friendly GUI was developed by using the Python Tkinter library. A case study using the PNNL taxonomy feeders R4-12.47-2 and smart grid network model with gateways was conducted to validate the utilized framework, thus demonstrating the range of potential applications of the framework. In addition, the functionality and user evaluations of GridAttackAnalyzer were carried out, with our results showing that users have a satisfying experience regarding the usability of GridAttackAnalyzer.

The architecture of GridAttackAnalyzer—Cyber Attack Analysis for Smart Grids is designed based on the general smart grid cybersecurity training’s analytical modeling approach. GridAttackAnalyzer takes the combination of the smart grid model, security settings, and database as the input to prepare the analysis session’s environment and calculates the security metrics via the employment of the preprocessing components to enable the analysis of various attack types. GridAttackAnalyzer is enhanced by recording all possible attack paths and computing the values of selected security metrics throughout the vulnerability analysis process. Furthermore, the attack graph can be generated automatically to capture attack paths.

The main contribution of our study is a framework that can effectively support realistic cybersecurity experimentation for the smart grid, with a focus on attack analysis. This framework was implemented in the form of GridAttackAnalyzer. Scientists can use the framework to estimate the effects of various attack types, as well as build and test early detection methods and mitigation strategies for anomalies even before they are implemented. The tool can be used for training on smart grid security and how vulnerabilities in IoT devices can affect smart grid security. It is also possible to use the framework for smart grid technology deployment, for example, to identify the communication needs for efficient device operation.

In addition, the system can be used for the cybersecurity training of IT experts and cybersecurity professionals. For instance, on the basis of the evaluation of various security metrics, IT professionals and cybersecurity experts can identify all possible pathways of attack. The most vulnerable devices in the paths to be protected can be identified in advance to prevent the most severe consequence. Besides, it is possible to compare the efficiency of particular device-level techniques deployed for various devices. The efficiency of the security strategies of the smart grid system can be calculated at the network level. Our work can also assist infrastructure designers in estimating the damage cost of the smart grid attack.

The source code of GridAttackAnalyzer was published and freely available for download via our GitHub page [43]. For future work, we intend to extend GriAttackAnalyzer to integrate more network models and power grid test feeders. For example, there are various other test feeders, such as EPRI Representative Feeders [44], PG&E Prototypical Feeders, Benchmark Models for Low-Voltage Distribution Feeders [45], Agent-Based Distribution Test Feeder with Smart Grid Functionality [46], and Test Feeder for DG Protection Analysis [47]. Additionally, we will undertake additional case studies utilizing a variety of smart grid attack types and CVEs to validate our extended framework. We also hope to gain more user feedback as we deploy this tool and invite a wider range of users.

## Figures and Tables

**Table 1:** The database structure of GridAttackAnalyzer.

Sub-Module	Object	Description
Smart Grid Model	ID	ID of the Smart Grid Model
	list_name	Name of the Smart Grid Model
	streets_and_houses	List of the streets and the corresponding houses
	description	Smart Grid Model description
Smart Grid Devices	ID	ID of the Smart Grid device
	device_name	Name of the Smart Grid device
	CVE_list	The CVE list of the Smart Grid device
	group	Group of the device (HAN, NAN, SCADA)
	description	Smart Grid device description
CVE List	ID	ID of the CVE
	description	CVE description
	CVSS_Base_Score_2.0	CVSS Base Score 2.0
	Impact_Subscore	Impact Subscore
	Exploitability_Subscore	Exploitability Subscore

**Table 2:** List of mathematical symbols in alphabetical order.

Symbol	Description
$ac_{ap}$	attack cost of an attack path
$ac_{g}$	attack cost of an inner node
$aim_{ap}$	attack impact of an attack path
$aim_{g}$	attack impact of an inner node
$at_{t}$	attack tree t
$p_{g}$	attack success probability for each inner node *g* in $at_{t}$
$p_{t}$	the root value in $at_{t}$
$p_{v}$	attack success probability value of a vulnerability
$r_{ap}$	attack risk of an attack path
$r_{g}$	attack risk of an inner node
$V_{g}$	the set of vulnerabilities under gate *g*
ac	attack cost
AC	the attack cost of the network
aim	attack impact
AIM	the attack impact of the network
g	inner node (gate)
*p*	attack success probability
P	the attack success probability of the network
*r*	attack risk
R	the attack risk of the network

**Table 3:** CVE list for smart grid devices.

No.	Smart Devices	CVE Lists
1	IP Camera	CVE-2020-11623, CVE-2020-11949, CVE-2020-3110
2	Smart TV	CVE-2020-9264, CVE-2019-12477, CVE-2019-11336, CVE-2019-9871, CVE-2018-13989
3	Smart Vacuum Cleaner	CVE-2019-12820, CVE-2019-12821, CVE-2018-20785, CVE-2018-17177, CVE-2018-10987
4	Smart Thermostat	CVE-2013-4860, CVE-2018-11315
5	Smart Light	CVE-2020-6007, CVE-2019-18980, CVE-2017-14797
6	Smart Meter	CVE-2017-9944
7	Gateway	CVE-2018-3880, CVE-2018-3879, CVE-2018-3902, CVE-2018-3909, CVE-2018-3907, CVE-2018-3911
8	Concentrator	CVE-2020-1638
9	FEP	CVE-2019-6810, CVE-2018-4838, CVE-2019-14813
10	ICCP Server	CVE-2015-6574, CVE-2006-0059
11	Communication Server	CVE-2021-20501, CVE-2020-7247, CVE-2020-27777
12	Local Terminal	CVE-2021-33200, CVE-2019-19816, CVE-2019-19814
13	Substation RTU	CVE-2019-14931, CVE-2020-7801, CVE-2019-16879, CVE-2019-20045
14	EMS/DRP Server	CVE-2020-9391, CVE-2019-6454, CVE-2019-14813

**Table 4:** The classification of attack paths based on the probability ranges adapted from [36,37].

Likelihood	Probability Ranges (p)
Almost Certain	0.8–1.0
Likely	0.6–0.79
Possible	0.4–0.59
Unlikely	0.2–0.39
Rare	0.0–0.19

**Table 5:** Attack analysis results.

Scenario	Entry Point	Patch	Security Metrics				Number of Paths					
			*p*	*c*	*aim*	*r*	Total	Rare	Unlikely	Possible	Likely	Almost Certain
1	Smart TV	No	1	21.7	33.9	33.9	16	0	3	0	5	8
2	Smart Thermostat	No	0.65	19.7	33.9	22.04	16	0	2	8	6	0
3	Robot Vacuum Cleaner	No	0.86	21.7	33.9	29.15	16	1	6	2	4	3
4	Smart Light	No	1	21.7	30.3	30.3	16	1	9	2	0	4
5	IP Camera	No	0.8	19.7	33.9	27.12	16	0	7	2	5	2
6	All	No	1	19.7	33.9	33.9	80	2	27	14	20	17
7	All	Smart TV	1	19.7	33.9	33.9	64	2	24	14	15	9
8	All	Smart TV and Smart Light	0.86	19.7	33.9	29.15	48	1	15	12	15	5
9 *	All	No	0.39	22.09	36.29	14.15	125	66	59	0	0	0
10	Substation (Local Terminal)	No	0.05	18.57	22.17	1.131	12	12	0	0	0	0

* Multiple-entry Multiple-target Attack Model.

**Table 6:** Functionality evaluation of GridAttackAnalyzer (a blank cell indicates a functionality/aspect that is not present).

No.	Year	Research	Attack Tree	Attack Graph		Security Metrics Calculation				Likelihood
				Attack Graph Generation	Attack Graph Visualization	Attack Success Probability	Attack Cost	Attack Impact	Attack Risk	
1	2011	Security Risk Analysis of Enterprise Networks Using Probabilistic Attack Graphs		Y		Y		Y	Y	
2	2011	Defining and Assessing Quantitative Security Risk Measures Using Vulnerability Lifecycle and CVSS Metrics				Y		Y	Y	
3	2012	Aggregating CVSS Base Scores for Semantics-Rich Network Security Metrics		Y				Y		
4	2012	Dynamic Security Risk Management Using Bayesian Attack Graphs	Y	Y		Y	Y		Y	
5	2014	Determining the Probability of Smart Grid Attacks by Combining Attack Tree and Attack Graph Analysis	Y	Y		Y				
6	2014	Attack Graph-Based Risk Assessment and Optimisation Approach	Y	Y		Y			Y	
7	2015	A Framework for Modeling and Assessing Security of the Internet of Things	Y	Y		Y	Y	Y	Y	
8	2016	Security Modelling and Analysis of Dynamic Enterprise Networks				Y	Y	Y	Y	
9	2017	A Quantitative CVSS-Based Cyber Security Risk Assessment Methodology For IT Systems		Y		Y		Y	Y	
*10*	*2017*	*A framework for automating security analysis of* *the internet of things*	*Y*	*Y*		*Y*	*Y*	*Y*	*Y*	
11	2018	A Comprehensive Analysis of Smart Grid Systems against Cyber-Physical Attacks	Y	Y		Y		Y	Y	Y
12	2019	CloudSafe: A Tool for an Automated Security Analysis for Cloud Computing		Y		Y				
13	2019	Quantitative Model of Attacks on Distribution Automation Systems Based on CVSS and Attack Trees	Y	Y		Y				
14	2020	A Bayesian Attack Tree Based Approach to Assess Cyber-Physical Security of Power System	Y	Y		Y		Y	Y	
15	2020	A Framework for Real-Time Intrusion Response in Software Defined Networking Using Precomputed Graphical Security Models		Y		Y	Y	Y	Y	
16	2021	Monitoring Cyber-Physical Layer of Smart Grid Using Graph Theory Approach	Y	Y						
17	2022	GridAttackAnalyzer	Y	Y	Y	Y	Y	Y	Y	Y

**Table 7:** SUS Results of GridAttackAnalyzer.

Framework	Maximum Value	Minimum Value	Mean	Standard Deviation
GridAttack-Analyzer	90	60	72.2	10.2

## Data Availability

Not applicable.

## Abbreviations

The following abbreviations are used in this manuscript:

AG	Attack Graph
AGG	Attack Graph Generation
AT	Attack Tree
CSV	Comma-Separated Values
CVE	Common Vulnerability Exposure
CVSS	Common Vulnerability Score System
DHS	U.S. Department of Homeland Security
DRP	Demand Response
EMS	Energy Management Systems
ENISA	European Union Agency for Cybersecurity
FEP	Front End Processor
GrSM	Graphical Security Model
HAN	Home Area Network
HARM	Hierarchical Attack Representation Model
ICS-CERT	U.S. Industrial Control Systems Cyber Emergency Response Team
IoT	Internet of Things
NAN	Neighbor Area Network
NVD	National Vulnerability Database
PNNL	Pacific Northwest National Laboratory
SCADA	Supervisory Control and Data Acquisition
SUS	System Usability Scale
WAN	Wide Area Network
